# Comparison of five influenza surveillance systems during the 2009 pandemic and their association with media attention

**DOI:** 10.1186/1471-2458-13-881

**Published:** 2013-09-24

**Authors:** Marit MA de Lange, Adam Meijer, Ingrid HM Friesema, Gé A Donker, Carl E Koppeschaar, Mariëtte Hooiveld, Nel Ruigrok, Wim van der Hoek

**Affiliations:** 1 National Institute for Public Health and the Environment (RIVM), Centre for Infectious Disease Control Netherlands, P.O. Box 1, 3720 BA Bilthoven, The Netherlands; 2 NIVEL, Netherlands Institute for Health Services Research, P.O. Box 1568, 3500 BN Utrecht, The Netherlands; 3 The Great Influenza Survey / Science in Action, P.O. Box 1786, 1000 BT Amsterdam, The Netherlands; 4 The Netherlands News Monitor, Buitenveldertselaan 3, 1082 VA Amsterdam, The Netherlands

**Keywords:** Influenza virus, Pandemic, Surveillance, Influenza-like illness, Media attention

## Abstract

**Background:**

During the 2009 influenza pandemic period, routine surveillance of influenza-like-illness (ILI) was conducted in The Netherlands by a network of sentinel general practitioners (GPs). In addition during the pandemic period, four other ILI/influenza surveillance systems existed. For pandemic preparedness, we evaluated the performance of the sentinel system and the others to assess which of the four could be useful additions in the future. We also assessed whether performance of the five systems was influenced by media reports during the pandemic period.

**Methods:**

The trends in ILI consultation rates reported by sentinel GPs from 20 April 2009 through 3 January 2010 were compared with trends in data from the other systems: ILI cases self-reported through the web-based Great Influenza Survey (GIS); influenza-related web searches through Google Flu Trends (GFT); patients admitted to hospital with laboratory-confirmed pandemic influenza, and detections of influenza virus by laboratories. In addition, correlations were determined between ILI consultation rates of the sentinel GPs and data from the four other systems. We also compared the trends of the five surveillance systems with trends in pandemic-related newspaper and television coverage and determined correlation coefficients with and without time lags.

**Results:**

The four other systems showed similar trends and had strong correlations with the ILI consultation rates reported by sentinel GPs. The number of influenza virus detections was the only system to register a summer peak. Increases in the number of newspaper articles and television broadcasts did not precede increases in activity among the five surveillance systems.

**Conclusions:**

The sentinel general practice network should remain the basis of influenza surveillance, as it integrates epidemiological and virological information and was able to maintain stability and continuity under pandemic pressure. Hospital and virological data are important during a pandemic, tracking the severity, molecular and phenotypic characterization of the viruses and confirming whether ILI incidence is truly related to influenza virus infections. GIS showed that web-based, self-reported ILI can be a useful addition, especially if virological self-sampling is added and an epidemic threshold could be determined. GFT showed negligible added value.

## Background

In April 2009, the first cases of influenza A(H1N1)pdm09 virus infection were confirmed in Mexico and the United States [1]. On 11 June 2009, the World Health Organization declared the first influenza pandemic of the 21st century [2]. In The Netherlands, the first case was confirmed on 30 April 2009 [3], and influenza activity was increased from 5 October through 13 December 2009 [4].

The Dutch Continuous Morbidity Registration sentinel general practice network was established in 1970 for surveillance of influenza-like illness (ILI). Following the 1992/1993 influenza season, virological investigation was added to the network [4]. Most European countries have comparable integrated epidemiological-virological sentinel surveillance systems [5]. However, even before the 2009 pandemic, the ability of these systems to cope with pressure during a pandemic was a matter of concern. There was discussion as to whether or not ancillary surveillance systems were needed in preparation for and during a pandemic [6]. During the 2009 pandemic in The Netherlands, four other surveillance systems were used. These included an internet-based monitor of self-reported ILI symptoms in the Dutch and Belgian general population: the Great Influenza Survey (GIS) [7,8]. Another was Google Flu Trends (GFT), that estimates ILI incidence based on influenza-related queries to online search engines [9]. A third system was the number of influenza virus detections reported by virology laboratories. Finally, during the pandemic period, all hospitals were required to report patients admitted due to laboratory-confirmed influenza A(H1N1)pdm09 virus infection [10,11].

Throughout the pandemic period in Europe, strong media coverage took place. In Wales, the first wave of ILI consultation rates reported by sentinel general practitioners (GPs) in 2009 was possibly influenced by the intensive media activity [12]. The media are known to be able to influence the behavior of the public as well as health professionals [13]. In the UK, media coverage may have biased estimates of ILI and influenza virus infections, as it created significant anxiety in the population [14]. In The Netherlands, coverage may have influenced information-seeking behavior on the internet, participation in web-based surveillance systems, healthcare-seeking behavior, and laboratory testing practices.

In preparation for a future influenza pandemic, it is important to evaluate how the Dutch sentinel general practice network and the four other surveillance systems performed during the pandemic period in 2009. Therefore, our aim was to assess the performance of the routine influenza surveillance system (ILI consultation rates reported by sentinel GPs) and whether the other available surveillance systems would be useful additions to the sentinel system. We also studied whether increased media coverage influenced the data trends of the five surveillance systems during the pandemic period.

## Methods

### Data sources

#### GP data

The sentinel general practice network covers a patient population which represents the national population in gender, age, regional distribution, and population density [4]. In 2009, the network included 42 sentinel practices that had a total of 129,065 enrolled patients in the average year. In June 2009, an additional 12 general practices accepted to join the network from the Netherlands Information Network of GPs care (LINH) and participated in surveillance for the duration of the pandemic [15]. The network defined ILI as sudden onset, fever (≥ 38.0°C) accompanied by cough, sore throat, running nose, frontal headache, retrosternal chest pain or muscle pain [4]. Incidence was defined as the weekly number of people who consulted their GP with ILI divided by the total number of patients enrolled in the sentinel practices. Increased influenza activity was defined as an ILI consultation rate higher than 5.1 per 10,000 persons for two consecutive weeks, accompanied by detection of influenza virus in respiratory specimens. Each general practice took a nose and throat swab from two ILI patients per week. Practices who saw no such patients in a given week were requested to swab two patients with another acute respiratory infection (ARI) [16]. To evaluate how well the sentinel general practice network functioned during the pandemic, we compared 2009 with the five preceding years (2004 through 2008) as to the percentage of days for which a sentinel group reported no ILI/influenza cases.

To estimate the workload of the GPs during the pandemic period, we accessed the electronic medical files of all 68 LINH GPs to obtain data on all their patient contacts by diagnosis and type of consultation: i.e. clinic visits, home visits, and telephone consultations [15]. All consultations for influenza, as defined by the International Classification of Primary Care code R80, were included and the weekly number was expressed per 10,000 enrolled patients.

Informed consent was not needed, as the Dutch Central Committee on Research Involving Human Subjects considers it not obligatory for routine surveillance studies using anonymous data. The privacy regulation of the LINH network was approved by the Dutch Data Protection Authority.

#### Great Influenza Survey (GIS)

This data was used with permission from the company Science in Action, which started GIS in the 2003/2004 influenza season. Since that season, yearly press releases have encouraged people from the Dutch and Belgian general population to fill in a web-based baseline questionnaire asking for demographical, medical and lifestyle data. Participants receive a weekly e-mail with a link to a short questionnaire asking about ILI symptoms experienced since their previous visit to the website. When symptoms are reported, additional questions are asked about GP consultations and whether or not daily activities were adjusted due to the symptoms. The GIS case definition of ILI is sudden onset of fever accompanied by muscle pain and cough and/or sore throat and/or chest pain. Measurement of body temperature is not required [8]. The incidence of ILI was defined as the weekly number of GIS participants reporting ILI symptoms divided by the total number of participants that week.

In our study, we excluded Belgian participants and selected the Dutch incidence data with certain restrictions. Cases of ILI were used only if reported within 2 weeks after day of onset. Cases were considered recurrent only when at least one week without symptoms fell between episodes. A person who participated in GIS more than once in a given week was counted only once for that week. Finally, to maximize data reliability, we sought the most active and experienced participants by 1) eliminating the first instance of GIS participation for each individual and 2) excluding any individual with fewer than three instances of participation. For the pandemic season of 2009, about 20,000 GIS participants fit these criteria and were used in our study.

#### Google Flu Trends (GFT)

GFT is a free internet-based surveillance tool which uses an automated method of selecting ILI/influenza-related search queries to estimate the ILI incidence. A search query is a complete, exact sequence of terms issued by a Google search user. Its weekly estimates reflect findings from Sunday through Saturday, whereas the other four surveillance systems and media coverage reflect findings from Monday through Sunday [9]. At http://www.google.org/flutrends (data openly available), we downloaded the estimated ILI incidence for The Netherlands for the period 20 April 2009 through 3 January 2010 [17]. No information was available on participants’ background, age or gender.

#### Hospital admissions

On 29 April 2009, influenza A(H1N1)pdm09 virus infection was designated a “category A notifiable disease” in The Netherlands. From that date, doctors and laboratories had to report the name of the patient to the Municipal Health Service (GGD) when the infection was suspected or identified. Each notification was then entered into a national anonymous and password-protected web-based database including information on the patient’s travel history, vaccination status, clinical symptoms, co-morbidity, treatments, hospitalisations and contact with symptomatic cases. On 15 August 2009, the notification criteria changed. From that date, reporting was mandated only for cases who were admitted to a hospital or died because of a laboratory-confirmed influenza A(H1N1)pdm09 virus infection [10,11]. The incidence per week was defined as the number of hospital admissions divided by the total population of The Netherlands in 2009.

As the National Institute for Public Health and the Environment (RIVM) is legally permitted to use these anonymous data, permission from a research ethics committee was not needed for our study.

#### Laboratory detections of influenza virus

Data on influenza virus detections was obtained from three sources.

From 20 April through 9 August 2009 all suspected influenza A(H1N1)pdm09 cases underwent virological testing at the National Influenza Center, located at the Erasmus Medical Center in Rotterdam, and at RIVM in Bilthoven. After 29 June through 9 August 2009, this effort was joined by nine regional outbreak assistance laboratories [18,19].

After 9 August 2009, not all suspected cases were virologically tested anymore. After this date, we therefore used data of The Dutch Working Group for Clinical Virology. This group issued its usual voluntary Weekly Virology Report, listing detections of influenza A and B virus at medical microbiological laboratories. These 21 laboratories around the country included the nine mentioned above.

Also for the total period (20 April 2009 through 3 January 2010), the sentinel GPs continued their normal routine by collecting nose and throat swabs for virus detection, as described above. The specimens were tested at the RIVM for the presence of influenza virus types A and B, and the influenza A viruses were further sub-typed [4].

The reporting week of the positive specimens of the first period (through 9 August) and those from the sentinel general practice network was defined as the week in which specimens were collected. The reporting week of positive specimens noted in the Weekly Virology Reports was the week of laboratory diagnosis. Because of the urgency for laboratory diagnosis during the pandemic period, those weeks are generally comparable.

All virological data described above are regularly provided by laboratories to RIVM as part of routine surveillance of influenza. Aggregated data are freely available at the RIVM website (in Dutch): http://www.rivm.nl/Onderwerpen/Onderwerpen/V/Virologische_weekstaten/Rapportages/Open_rapportages_virologische_weekstaten and http://www.rivm.nl/griep.

#### Media attention

The Netherlands News Monitor (NNM), an independent scientific institute researching journalism in The Netherlands, provided data on media attention. Its researchers screened newspaper articles and television broadcasts related to the influenza pandemic [20]. They included national newspapers that are distributed free of charge five times a week (‘Metro’, ‘Spits’, ‘de Pers’) and subscription-based national newspapers distributed six times a week (‘NRC Handelsblad’, ‘Algemeen Dagblad’, ‘de Volkskrant’, ‘Trouw’, ‘De Telegraaf’). These eight newspapers had a total of 2,964,364 daily copies [21]. For comparison, there were 7 million households in The Netherlands on 1 January 2009 [22]. For our study, we selected all articles about the influenza that appeared from 20 April 2009 through 3 January 2010.

In addition, NNM screened daily news broadcasts on national television that discussed pandemic influenza. These included the ‘NOS Journaal’ (aired at 8 pm), ‘RTL Nieuws’ (7.30 pm) and ‘Hart van Nederland’ (7 pm) [20]. The first two programs had most viewers, averaging 3.4 and 1.4 million. The third program averaged about 1 million but was included because its audience was considered to be different from the audience of the other two [23]. The Dutch population consisted of 16 million people on 1 January 2009 [22]. Our study focused on all influenza-related broadcasts that aired from 20 April through 3 January 2010.

For each newspaper or television report, NNM determined the source from the content of a sample of all newspapers articles and television broadcasts. The source was defined as the person or authority that was cited in the media report [20].

### Statistical analysis

Weekly ILI consultation rates reported by the sentinel GPs and ILI/influenza data from the four other surveillance systems were compared, and Spearman rank correlation coefficients were determined for the period 20 April 2009 through 3 January 2010.

To assess whether the media reports influenced the ILI/influenza data reported by the five surveillance systems, the data are graphically displayed and compared for the period 20 April 2009 through 3 January 2010. In addition, Spearman rank correlation coefficients were determined between the weekly ILI/influenza rates and the weekly number of newspaper articles and television broadcasts for the same period. Furthermore, Spearman rank correlation coefficients were determined for the periods in which media attention was observed to coincide with the trends of the ILI/influenza surveillance systems. Time lags and 95% confidence intervals of the correlation coefficients were calculated to investigate if the media attention preceded rises in ILI/influenza rates.

Statistical significance was set at p < 0.05. All data were analysed using SAS version 9.2 (SAS Institute Inc., USA).

## Results

### Trends in ILI/influenza

**Figure 1:**
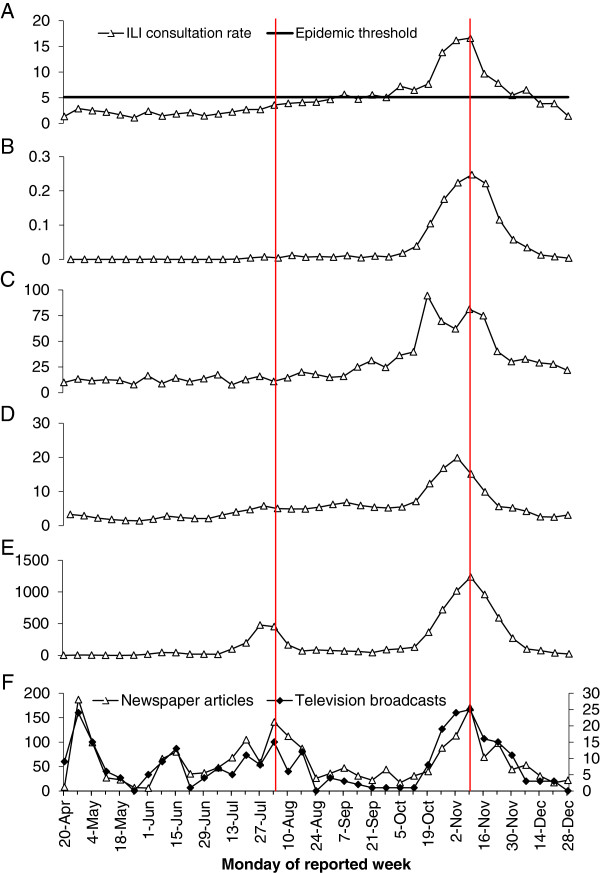
**Trends in media attention and ILI/influenza rates in 2009. A**. ILI/influenza consultation rates reported by the sentinel general practices per 10,000 enrolled patients. **B**. Number of hospitalisations for influenza type A(H1N1)pdm09 infection per 10,000 inhabitants. **C**. ILI incidence, Great Influenza Survey, per 10,000 participants. **D**. Estimated ILI incidence, Google Flu Trends, per 10,000 inhabitants. **E**. Total number of influenza virus detections. **F**. Number of newspaper articles (left axis) and number of television broadcasts (right axis) related to pandemic influenza. The vertical red lines indicate peaks of media attention which coincided with the peaks of ILI/influenza data reported by surveillance systems.

The ILI consultation rates reported by the sentinel GPs (Figure 1A) and the incidence of hospital admissions due to influenza A(H1N1)pdm09 (Figure 1B) both peaked in the week of 9 November 2009. In the year 2009, the percentage of non-reporting days was not higher (9.0%) compared to the average percentage during the 5 years before the pandemic (13.2%) [4]. The consultation rates of the sentinel GPs, indicated increased influenza activity from 5 October through 13 December 2009. Likewise, an average of 36.8% of sentinel-submitted samples tested positive for influenza virus, which was higher than seen before or after this period. The incidence of hospital admissions also showed a continuous increase from 5 October 2009 onwards. The GIS ILI incidence increased from 5 October 2009 onwards (Figure 1C) and peaked in the weeks of 26 October and 9 November 2009. The incidence according to GFT started to rise one week later, on 12 October 2009, and peaked in the week of 2 November 2009 (Figure 1D). The number of influenza virus detections (Figure 1E) had two peaks: a small summer peak in the weeks of 27 July and 3 August and a second increase from the week of 19 October onwards with a peak in the week of 9 November 2009. Both peaks coincide with peaks of the number of newspapers and television broadcasts.

From 20 April 2009 through 3 January 2010, only 26 cases of influenza virus type B were noted in the Weekly Virology Report of hospital laboratories in The Netherlands. Therefore, almost all their influenza virus detections were type A (n = 6,802). Because almost all sub-typed influenza A viruses (99.7%) obtained from sentinel GPs were A(H1N1)pdm09, we assume that the clinical observations at both sentinel practices and hospitals represented A(H1N1)pdm09 influenza virus activity.

### Distribution of GP visitors and GIS participants

The ILI consultation rates of the sentinel general practice network were highest for children 0–4 years old, followed by those 5–14 years old. The lowest ILI consultation rate was seen for people 65 years of age or older (data not shown).

Most of the GIS participants were 15 to 64 years old (91.5%), and the majority were female (66.1%).

### Type of GP consultations

**Figure 2:**
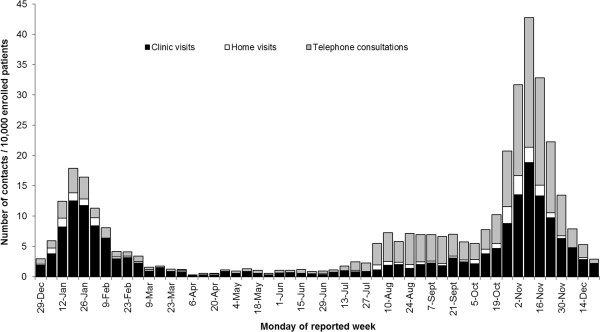
General practitioner-patient contacts for influenza in The Netherlands Information Network of General Practice (LINH) during the year 2009.

Figure 2 displays the type of ILI/influenza consultations with sentinel GPs that occurred from 1 January 2009 through 31 December 2009. The number of clinic and home visits showed only a slight increase compared to the 2008/2009 influenza season, but the number of telephone consultations was much higher during the pandemic period, comprising half the total. For comparison, during the peak of the 2008/2009 influenza season, 23% of all contacts were telephone consultations.

### Trends in media attention

In the period from 20 April 2009 through 3 January 2010, 2,194 newspaper articles and 294 television broadcasts were counted with pandemic flu as a topic. Figure 1F displays the weekly number of articles and broadcasts about influenza virus A(H1N1)pdm09. The number of newspaper articles peaked several times: in the week of 27 April, 15 June, 20 July, 3 August and 9 November 2009. Television broadcasts peaked in roughly the same weeks: 27 April, 15 June, 3 August, and 9 November 2009.

The most frequently quoted sources in media reports about pandemic influenza were from the general society (e.g. business representatives and ordinary citizens), the government, and experts such as RIVM and NIVEL (National Institute for Health Services Research) [20].

### Correlations

All four other surveillance systems (GIS, GFT, hospital admissions, and laboratory detections of influenza) had a strong correlation with the ILI consultation rates of the sentinel GPs (Table 1). The highest correlation coefficient was seen for the incidence of hospital admissions, followed by the ILI incidence estimated by GFT and reported by GIS, respectively.

**Table 1:** Correlation between ILI consultation rates of the sentinel general practice network and four other surveillance systems^*^

	**Number of hospital admissions/10,000 inhabitants**	**ILI incidence GIS/10,000 participants**	**Estimated ILI incidence GFT/10,000 inhabitants**	**Number of influenza virus detections**
ILI consultation rates, sentinel GPs/10,000 enrolled patients	0.92	0.84	0.84	0.77
	(p < 0.001)	(p < 0.001)	(p < 0.001)	(p < 0.001)

*ILI* influenza-like illness, *GIS* Great Influenza Survey, *GP* general practitioner, *GFT* Google Flu Trends.

*Spearman rank correlation coefficients were determined for the complete study period (20-4-2009 through 3-1-2010).

The correlation between trends in ILI/influenza rates and trends in media attention was low to moderate in the period of 20 April 2009 through 3 January 2010 (Table 2). The highest correlation coefficient was seen for the number of influenza virus detections compared to both the number of newspaper articles (rho = 0.52) and the number of television broadcasts (rho = 0.36). These correlations were both statistically significant (p < 0.05). The lowest correlation was seen for the ILI incidence of GIS with both media types. The correlations between media attention and the five surveillance methods in the peak period (31 August 2009 through 3 January 2010) were stronger compared to the whole period (Table 3). The 95% confidence intervals of the correlation coefficients with one-week time lag overlapped those of the same week (data not shown). This result was seen for the peak in media attention in the period of 29 June 2009 through 23 August 2009 and for the peak in the period 31 August 2009 through 3 January 2010.

**Table 2:** Correlation between five influenza surveillance systems and media attention, 20-4-2009 – 3-1-2010*

**Surveillance system**	**Number of newspaper articles**		**Number of television broadcasts**	
	**Spearman rank correlation coefficient**	**P-value**	**Spearman rank correlation coefficient**	**P-value**
ILI consultation rates, sentinel GPs/10,000 enrolled patients	0.32	0.055	0.22	0.188
Number of hospital admissions/10,000 inhabitants	0.22	0.183	0.15	0.372
ILI incidence, GIS/10,000 participants	0.06	0.706	0.09	0.585
Estimated ILI incidence, GFT/10,000 inhabitants	0.34	0.039	0.23	0.162
Number of influenza virus detections	0.52	<0.001	0.36	0.030

*ILI* influenza-like illness, *GIS* Great Influenza Survey, *GP* general practitioner, *GFT* Google Flu Trends.

*Spearman rank correlation coefficients were determined for the complete study period (20-4-2009 through 3-1-2010).

**Table 3:** Correlation between five influenza surveillance systems and media attention, 31-8-2009 – 3-1-2010*

	**Number of newspaper articles**		**Number of television broadcasts**	
	**Spearman correlation coefficient**	**P-value**	**Spearman correlation coefficient**	**P-value**
ILI consultation rates, sentinel GPs/10,000 enrolled patients	0.74	<0.001	0.68	0.002
Number of hospital admissions/10,000 inhabitants	0.74	<0.001	0.80	<0.001
ILI incidence, GIS/10,000 participants	0.49	0.038	0.59	0.010
Estimated ILI incidence, GFT/10,000 inhabitants	0.62	0.006	0.61	0.007
Number of influenza virus detections	0.79	<0.001	0.79	<0.001

*ILI* influenza-like illness, *GIS* Great Influenza Survey, *GP* general practitioner, *GFT* Google Flu Trends.

*Spearman rank correlation coefficients were determined for the period in which media attention coincided with trends of the ILI/influenza surveillance systems (31-8-2009 through 3-1-2010).

## Discussion

### Key findings and interpretation

The sentinel general practice network maintained its stability and continuity during the pandemic in 2009, as GPs continued to report their weekly ILI consultation numbers and provide valid information for action. However, even in this relatively mild pandemic, their workload was higher than in the 2008–2009 influenza season, mainly due to an increased number of telephone consultations and extra influenza vaccinations.

All five ILI/influenza surveillance systems showed the same trends, with a strong correlation between the routinely used sentinel general practice network and the four other systems. However, the number of influenza virus detections was the only surveillance data that registered a summer peak in the week of 3 August 2009. This was the result of a policy of intensive case-finding and the return of infected travellers during that period. Probably because effective transmission had not been established at the time, the other systems did not detect this summer peak.

From this study, it is hard to conclude which other system gave an earlier signal of increased influenza activity in the pandemic peak period than the ILI consultation rates reported by the sentinel GPs, because the trends were very similar, and a mathematically derived epidemic threshold was available only for the sentinel general practice network. No surveillance system gave an earlier increase in the pandemic period compared to the increased influenza activity determined by the sentinel GPs. Although, in the seasons 2003/2004 through 2007/2008 the trend in GIS-ILI incidence was one week ahead compared to the ILI consultation rates reported by the sentinel GPs [8].

Since all five ILI/influenza surveillance systems showed the same trends during the 2009 pandemic period, which of the other systems would be a useful addition to the sentinel general practice network during a future pandemic? Information about hospital admissions is a very important addition, providing information about the severe cases. Virological data is likewise valuable, being needed to confirm whether the ILI incidence is truly related to influenza virus infections, to monitor for virulence and resistance markers, to monitor for possible changes in immune-dominant regions, and to estimate the efficacy of current vaccines. With regard to recommendations of strains for future vaccines, antigenic characterization data is important to understand the match of circulating viruses with the current vaccine strain. The GIS system could be a useful addition, because it measures the ILI incidence directly in the community. Additionally, it is a cheap and flexible system and collects a lot of background information about participants. Its value will increase if it adds virological testing, thus combining epidemiological and virological data. The GFT system, however, adds no information compared to the other systems and reveals nothing about its users or whether they truly had an influenza infection.

According to the agenda-setting theory, newspapers and broadcasts select certain topics and omit certain topics. This selective coverage influences what subjects the audience knows about, thinks about, and has feelings about [24,25]. The influenza pandemic turned out to be a big topic in the news media at various points in the period from 20 April 2009 through 3 January 2010, with an important potential impact on the public agenda and the surveillance data. While causal inference cannot be inferred, this study provides no indication that media attention preceded increasing trends in ILI incidence and influenza virus detections as reported in five surveillance systems during the pandemic period. We found that the number of newspaper articles and television broadcasts peaked more often compared to the surveillance data. Correlations between the five surveillance systems and media attention were low-moderate in the pandemic period covering 20 April 2009 through 3 January 2010, albeit stronger in the peak pandemic period from 31 August 2009 through 3 January 2010. Even in that peak period, media coverage did not peak earlier than data from surveillance systems. The 95% confidence intervals of the correlation coefficients between the media attention and the five surveillance systems with one-week time lag did overlap those of the same week. Finally, there was no indication that media attention preceded the number of laboratory detections of influenza at its summer peak (29 June 2009 through 23 August 2009).

In the intensive case finding period of the pandemic (till 9 august 2009), weekly reports of laboratory detections of cases were presented on Fridays on the website of the RIVM (http://www.rivm.nl). Therefore, the summer peak in media attention probably reflects the weekly reports of the case finding as reflected in the number of A(H1N1)pdm09-positive specimens (Figure 1E).

### Comparison to other studies

The comparability between traditional surveillance systems (ILI consultation rates reported by sentinel GPs) and newer surveillance systems (GIS and GFT) was established in several countries in non-pandemic years [7-9,26,27]. Our research and other studies confirm that this comparability also existed during the pandemic period [28-32].

The findings of our study are in line with those from New Zealand where trends of media attention peaked more often than trends of GFT-estimated ILI incidence and consultation rates reported by the national sentinel general practice network during the pandemic period [28]. In Guam, similar to our study, a high correlation was found between the temporal pattern in number of ‘swine flu’ stories and the temporal pattern in clinical diagnoses of ARI in the emergency department of a hospital and laboratory-confirmed cases of influenza A(H1N1)pdm09. Unlike our study, the number of ‘swine flu’ stories peaked one week earlier compared to the hospital emergency room ARI data, suggesting media influence on consultation behavior. However, no correlation coefficients with time lag were determined in that study [33]. In the UK, newspaper coverage about A(H1N1)pdm09 likewise increased one week before increases in the number of laboratory-confirmed cases in the beginning of the pandemic period [34].

### Limitations

Several limitations were inherent to the surveillance systems examined in this study. For example, the sentinel general practice network mostly captured young children, both during the pandemic and in the seasons before [35], probably because young children are brought to the doctor by concerned parents. During the pandemic, GIS-ILI cases were not virological confirmed, although it began to address this limitation in the 2011/2012 influenza season, requiring the GPs in Belgium to swab patients and also starting a pilot program in Sweden that includes self-sampling. Moreover, in this study, GIS participants did not represent the Dutch general population, with 91.1% of participants being 15 to 64 years of age whereas 67.3% of the Dutch population falls in this age group. In addition, more women than men participated than are represented in the general population (67.3% versus 50.5%) [36]. During the years 2003 through 2008, children and the elderly were underrepresented in GIS; its elderly population was healthier, participants aged 15–24 years were less often employed, and participants under 45 had a higher vaccination uptake compared to the Dutch population [8]. Like GIS, GFT is prone to selective participation and in addition lacks virological confirmation.

Besides the limitations inherent to the surveillance systems, other limitations were associated with our study. We only counted the newspaper articles and television broadcasts about influenza A(H1N1)pdm09 which had been collected in context of another study [20]. Beyond articles and broadcasts, people could have obtained information about the pandemic from other sources, such as social media [37,38]. As an emerging source of information, social media was hypothesized to have limited impact on our study during the pandemic period, but it should be monitored in future studies. An epidemic threshold was only determined for the ILI consultation rates reported by the sentinel GPs [39], and its determination for the other surveillance systems would possibly assess the period of increased influenza activity in an earlier way. A final limitation of this study is that there was no daily data available for most of the surveillance systems. Therefore, we could not investigate the influence of the media attention on a daily basis.

## Conclusions

Results of different surveillance systems during the influenza pandemic showed similar trends and were highly correlated with each other. There was no indication that media attention influenced the trends in ILI/influenza rates. The number of virus detections was the only system which registered a summer peak, thus giving the earliest signal. The ILI consultation rates reported by the sentinel GPs remain the basis of surveillance in The Netherlands, because the system integrates epidemiological and virological information and was able to sustain its operation under pandemic pressure. Hospital data and virological data will remain very important during a pandemic period, providing information on the severity, molecular and phenotypic characterization of the viruses, and whether the ILI incidence is truly related to influenza virus infections. GIS can be a good addition during a pandemic period, because it is a cheap and flexible method and provides a lot of background information about the participants. GIS will be even more useful when it includes virological testing and when determination of an epidemic threshold can help to detect an impending epidemic. GFT showed negligible added value.

## Abbreviations

ARI: Acute respiratory infection; GFT: Google flu trends; GGD: Municipal health services; GIS: Great influenza survey; GP: General practitioner; ILI: Influenza-like illness; LINH: Netherlands information network of GPs care; NIVEL: Netherlands institute for health services research; NNM: Netherlands news monitor; RIVM: National institute for public health and the environment; SAS: Statistical analysis software.

## Competing interests

The authors declare that they have no competing interests.

## Authors’ contributions

MMAdL contributed to the design of the study, performed the statistical analysis, and drafted the manuscript. WvdH contributed to the design of the study and helped to draft the manuscript. IHMF assisted with the statistical analysis and reviewed the article critically. AM coordinated the virus diagnostics, provided help with the data collection and revised the article. GAD, MH, NR and CEK collected data and reviewed the article critically. All authors read and approved the final manuscript.

## Pre-publication history

The pre-publication history for this paper can be accessed here:

http://www.biomedcentral.com/1471-2458/13/881/prepub
